# ﻿Morphological and molecular identification for four new wood-inhabiting species of *Trechispora* (Basidiomycota) from China

**DOI:** 10.3897/mycokeys.105.120438

**Published:** 2024-05-15

**Authors:** Kai-Yue Luo, Jiang-Qing Su, Chang-Lin Zhao

**Affiliations:** 1 The Key Laboratory of Forest Resources Conservation and Utilization in the Southwest Mountains of China Ministry of Education, Key Laboratory of National Forestry and Grassland Administration on Biodiversity Conservation in Southwest China, Yunnan Provincial Key Laboratory for Conservation and Utilization of In-forest Resource, Southwest Forestry University, Kunming 650224, China Southwest Forestry University Kunming China; 2 College of Forestry, Southwest Forestry University, Kunming 650224, China Southwest Forestry University Kunming China

**Keywords:** East Asia, macrofungi, molecular systematics, taxonomy, 4 new taxa

## Abstract

Four new wood-inhabiting fungi, *Trechisporaalbofarinosa*, *T.bisterigmata*, *T.pileata* and *T.wenshanensis***spp. nov.**, are proposed based on a combination of morphological features and molecular evidence. *Trechisporaalbofarinosa* is characterized by the farinose basidiomata with flocculence hymenial surface, a monomitic hyphal system with clamped generative hyphae, and ellipsoid, warted basidiospores. *Trechisporabisterigmata* is characterized by the membranous basidiomata with odontioid hymenial surface, rhizomorphic sterile margin, barrelled basidia and subglobose to broad ellipsoid, smooth basidiospores. *Trechisporapileata* is characterized by the laterally contracted base, solitary or imbricate basidiomata, fan shaped pileus, radially striate-covered surface with appressed scales, odontioid hymenophore surface, and subglobose to broad ellipsoid, thin-walled, smooth basidiospores. *Trechisporawenshanensis* is characterized by a cottony basidiomata with a smooth hymenial surface, and ellipsoid, thin-walled, warted basidiospores. Sequences of ITS and LSU marker of the studied samples were generated, and phylogenetic analyses were performed with the maximum likelihood, maximum parsimony, and Bayesian inference methods. The phylogenetic tree inferred from the ITS+nLSU sequences highlighted that four new species were grouped into the genus *Trechispora*.

## ﻿Introduction

Fungi represent one of the most diverse groups of organisms on earth, with an indispensable role in the processes and functioning of ecosystems (Wu et al. 2020, 2021; Wang et al. 2021; Hyde 2022; Guan and Zhao 2023). Wood-inhabiting fungi play an important role in the carbon cycle (Dai et al. 2015; Wu et al. 2017; Chen and Zhao 2020; Guan et al. 2021; Liu et al. 2022a, b; Luo and Zhao 2022; Mardones et al. 2023; Yuan et al. 2023; Zhang et al. 2023; Zhao et al. 2023a, b; Zhou et al. 2023). The wood-inhabiting fungal order Trechisporales K.H. Larss. is a species-poor order, compared with most other orders within Agaricomycetes, Basidiomycota (Wijayawardene et al. 2022).

*Trechispora* P. Karst. (Hydnodontaceae) typified by *T.onusta* P. Karst., which is characterized by resupinate to effused basidiomata; a smooth to hydnoid to poroid hymenophore; ampullaceous septa; short cylindric basidia; and smooth to verrucose or aculeate basidiospores (Karsten 1890; Bernicchia and Gorjón 2010). Currently, MycoBank and Index Fungorum have registered 163 recorded and 156 recorded intraspecific names in *Trechispora*, respectively. About 100 species are currently accepted in *Trechispora* worldwide (Karsten 1890; Bondartsev and Singer 1941; Rogers and Jackson 1943; Rogers 1944; Bondartsev 1953; Liberta 1966, 1973; Parmasto 1968; Burdsall and Gilbertson 1982; Gilbertson and Budington 1970; Jülich 1975, 1976; Ryvarden 1975; Ryvarden and Liberta 1978; Hallenberg 1978, 1980; Jülich and Stalpers 1980; Rauschert 1987; Vries 1987; Larsson 1992, 1994, 1995, 1996; Hjortstam and Larsson 1995; Ryvarden 2002; Trichies and Schultheis 2002; Ryvarden et al. 2003; Miettinen and Larsson 2006; Dai 2011; Yuan and Dai 2012; Ordynets et al. 2015; Phookamsak et al. 2019; Xu et al. 2019; Chikowski et al. 2020; Haelewaters et al. 2020; Crous et al. 2021; de Meiras-Ottoni et al. 2021; Zhao and Zhao 2021; Liu et al. 2022a, b; Luo and Zhao 2022; Sommai et al. 2023), of which 38 species of the genus have been found in China (Dai 2011; Yuan and Dai 2012; Xu et al. 2019; Dai et al. 2021; Zhao and Zhao 2021; He et al. 2022; Liu et al. 2022a, b; Luo and Zhao 2022; Deng et al. 2023; Liu et al. 2024a, b).

There have been many studies on the phylogeny of this genus in recent years. A high phylogenetic diversity on the corticioid Agaricomycetes based on two genes, 5.8S and 28S showed that nine taxa of *Trechispora* nested into trechisporoid clade (Larsson et al. 2004). The molecular systematics suggested that *Trechispora* belonged to Hydnodontaceae and was related to genera *Brevicellicium* K.H. Larss. & Hjortstam, *Porpomyces* Jülich, *Sistotremastrum* J. Erikss., and *Subulicystidium* Parmasto (Telleria et al. 2013). Based on the ITS and nLSU datasets, the phylogenetic study of *Trechispora* reported two new *Trechispora* species as *T.cyatheae* Ordynets, Langer & K.H. Larss. and *T.echinocristallina* Ordynets, Langer & K.H. Larss., on La Réunion Island (Ordynets et al. 2015). The phylogeny of Trechisporales was inferred from a combined ITS-nLSU sequences, which revealed that two related genera *Porpomyces*, *Scytinopogon* Singer, grouped closely together with *Trechispora* and all of them nested within Hydnodontaceae (Liu et al. 2019). Based on ITS dataset, the three new species of *Trechispora* were described and used to evaluate the phylogenetic relationship with other species of this genus, in which *T.murina* was retrieved as a sister to *T.bambusicola* with moderate supports, and *T.odontioidea* formed a single lineage and then grouped with *T.fimbriata* and *T.nivea*, while *T.olivacea* formed a monophyletic lineage with *T.farinacea*, *T.hondurensis*, and *T.mollis* (Luo and Zhao 2022). Recently, based on the morphological features and molecular evidence, three new species of *Trechispora* have been reported from Northern and Northeastern Thailand (Sommai et al. 2023).

During investigations into the wood-inhabiting fungi in the Yunnan-Guizhou Plateau of China, samples representing four additional species belonging to genus *Trechispora* were collected. To clarify the placement and relationships of the four species, we carried out a phylogenetic and taxonomic study on *Trechispora*, based on the ITS+nLSU.

## ﻿Materials and methods

### ﻿Morphology

The specimens studied were deposited at the herbarium of Southwest Forestry University (SWFC), Kunming, Yunnan Province, China. The macromorphological descriptions were based on field notes and photos captured in the field and laboratory. Color, texture, taste and odor of basidiomata were mostly based on authors’ field trips. Color terminology followed Kornerup and Wanscher (1978). All materials were examined under a Nikon 80i microscope. Drawings were made with the aid of a drawing tube. The measurements and drawings of the microscopic structures were made (Wu et al. 2022). The following abbreviations were used: KOH = 5% potassium hydroxide water solution, CB = cotton blue, CB– = acyanophilous, IKI = Melzer’s reagent, IKI– = both inamyloid and indextrinoid, L = spore length (arithmetic average for all spores), W = spore width (arithmetic average for all spores), Q = L/W ratios of the specimens studied, and n = a/b (a = total number of spores measured, from b = number of specimens).

### ﻿Molecular phylogeny

The CTAB rapid plant genome extraction kit-DN14 (Aidlab Biotechnologies Co., Ltd, Beijing) was used to obtain genomic DNA from the dried specimens following the manufacturer’s instructions (Zhao and Wu 2017). The nuclear ribosomal ITS region was amplified with the primers ITS5 and ITS4 (White et al. 1990). The nuclear ribosomal LSU gene was amplified with the primers LR0R and LR7 (Vilgalys and Hester 1990; Rehner and Samuels 1994). The PCR procedure for ITS was as follows: initial denaturation at 95 °C for 3 min, followed by 35 cycles at 94 °C for 40 s, 58 °C for 45 s and 72 °C for 1 min, and a final extension of 72 °C for 10 min. The PCR procedure for nLSU was as follows: initial denaturation at 94 °C for 1 min, followed by 35 cycles at 94 °C for 30 s, 48 °C for 1 min and 72 °C for 1.5 min, and a final extension of 72 °C for 10 min. The PCR products were purified and directly sequenced at Kunming Tsingke Biological Technology Limited Company, Yunnan Province, China. All newly-generated sequences were deposited in NCBI GenBank (Table 1).

**Table 1.** List of species, specimens and GenBank accession numbers of sequences used in this study.

Species name	Specimen No.	GenBank accession No.		References
		ITS	LSU	
*Fibrodontiaalba*	TNM F24944	NR153983	NG060401	Yurchenko and Wu 2014
*F.brevidens*	Wu 9807-16	KC928276	KC928277	Yurchenko and Wu 2014
*Trechisporaalba*	CH21384	OR557258	–	Liu et al. 2024a
*T.albofarinosa*	CLZhao 4356	OQ241383	OQ282703	This study
*T.amianthina*	CBS 202.54	–	MH868822	Vu et al. 2019
*T.araneosa*	KHL 8570	AF347084	–	Larsson et al. 2004
*T.bambusicola*	CLZhao 3302	MW544021	MW520171	Zhao and Zhao 2021
*T.bambusicola*	CLZhao 3305	MW544022	MW520172	Zhao and Zhao 2021
*T.bispora*	CBS 142.63	MH858241	MH869842	Larsson et al. 2004
*T.bisterigmata*	CLZhao 2522	OQ241386	–	This study
*T.bisterigmata*	CLZhao 7870	OQ241387	–	This study
*T.byssinella*	UC 2023068	KP814481	–	Unpublished
*T.chartacea*	FLOR56185	MK458775	–	Liu et al. 2022a
*T.clancularis*	FRDBI 4426619	MW487976	–	Unpublished
*T.cohaerens*	HHB-19445	MW740327	–	Unpublished
*T.copiosa*	AMO427	MN701015	MN687973	de Meiras-Ottoni et al. 2021
*T.copiosa*	AMO450	MN701017	MN687974	de Meiras-Ottoni et al. 2021
*T.crystallina*	LWZ 20170729-2	OM523419	OM339238	Liu et al. 2022a
*T.cyatheae*	FR0219443	UDB024016	UDB024017	Ordynets et al. 2015
*T.cyatheae*	FR0219446	UDB024020	UDB024021	Ordynets et al. 2015
*T.dentata*	Dai 22565	OK298491	OM049408	Liu et al. 2022b
*T.dimitiella*	Dai 21181	OK298493	OK298949	Liu et al. 2022b
*T.dimitiella*	Dai 21931	OK298492	OK298948	Liu et al. 2022b
*T.echinospora*	E11/37-10	JX392850	JX392851	Telleria et al. 2013
*T.echinospora*	E11/37-12	JX392853	JX392854	Telleria et al. 2013
*T.farinacea*	356	AF347089	–	Larsson et al. 2004
*T.farinacea*	MA-Fungi 79474	JX392855	JX392856	Telleria et al. 2013
*T.fimbriata*	CLZhao 7969	MW544024	MW520174	Zhao and Zhao 2021
*T.fimbriata*	CLZhao 9006	MW544025	MW520175	Zhao and Zhao 2021
*T.foetida*	FLOR 56315	MK458769	–	Liu et al. 2022a
*T.fragilis*	Dai 20535	OK298494	OK298950	Liu et al. 2022b
*T.gelatinosa*	AMO824	MN701020	MN687977	de Meiras-Ottoni et al. 2021
*T.gelatinosa*	AMO1139	MN701021	MN687978	de Meiras-Ottoni et al. 2021
*T.gracilis*	LWZ 20170814-17	OM523435	OM339253	Liu et al. 2022a
*T.havencampii*	DED8300	NR154418	NG059993	Desjardin and Perry 2015
*T.hondurensis*	HONDURAS19-F016	NR178152	NG081479	Haelewaters et al. 2020
*T.hondurensis*	HONDURAS19-F016a	MT571523	MT636540	Haelewaters et al. 2020
*T.hymenocystis*	KHL 8795	AF347090	–	Unpublished
*T.hymenocystis*	KHL 16444	MT816397	–	Unpublished
*T.incisa*	GB0090521	KU747093	KU747086	Unpublished
*T.incisa*	GB0090648	KU747095	KU747087	Unpublished
*T.invisitata*	5425_537	ON963772	–	Unpublished
*T.invisitata*	UC2023088	KP814425	–	Unpublished
*T.kavinioides*	KGN 981002	AF347086	–	Unpublished
*T.laevispora*	Dai 21655	OK298495	OM108710	Liu et al. 2022b
*T.larssonii*	LWZ 20190817-11a	OM523442	OM339259	Liu et al. 2022a
*T.longiramosa*	HG 140168	OM523448	OM339264	Liu et al. 2022a
*T.mellina*	URM85756	–	MH280000	Unpublished
*T.microspora*	FRDBI 18772216	OL828778	–	Unpublished
*T.mollis*	URM85884	MK514945	MK514945	Unpublished
*T.mollis*	URM85885	–	MT423667	Unpublished
*T.mollusca*	iNAT 30809943	MZ269232	–	Unpublished
*T.mollusca*	CFMR:DLL2011-186	KJ140681	–	Unpublished
*T.nivea*	MA-Fungi 76238	JX392824	JX392825	Telleria et al. 2013
*T.nivea*	MA-Fungi 76257	JX392826	JX392827	Telleria et al. 2013
*T.pallescens*	FLOR56184	MK458767	–	Unpublished
*T.pallescens*	FLOR56188	MK458774	–	Unpublished
*T.papillosa*	AMO713	MN701022	MN687979	de Meiras-Ottoni et al. 2021
*T.papillosa*	AMO795	MN701023	MN687981	de Meiras-Ottoni et al. 2021
*T.patawaensis*	VPapp-GF1901	OL314550	OL314546	Unpublished
*T.perminispora*	LWZ2019081639a	OM523525	OM339329	Liu et al. 2024a
*T.pileata*	CLZhao 4456	OQ241388	OQ282715	This study
*T.praefocata*	FRDBI 18819116	OL828784	–	Unpublished
*T.regularis*	KHL 10881	AF347087	–	Unpublished
*T.rigida*	URM85754	MT406381	MH279999	Unpublished
*T.sinensis*	LWZ 20170816-35	OM523479	OM339287	Liu et al. 2022a
*T.stellulata*	14153	MW023104	–	Unpublished
*T.stellulata*	33962903	ON364078	–	Unpublished
*T.stellulata*	UC2023099	KP814451	–	Unpublished
*T.stellulata*	UC2023230	KP814491	–	Unpublished
*T.stevensonii*	MA-Fungi 70669	JX392841	JX392842	Telleria et al. 2013
*T.stevensonii*	MA-Fungi 70645	JX392843	JX392844	Telleria et al. 2013
*T.subfarinacea*	LWZ2020092133a	OM523528	OM339331	Liu et al. 2024a
*T.subhelvetica*	7089	JN710601	–	Unpublished
*T.subhymenocystis*	LWZ 20190818-29b	OM523492	OM339299	Liu et al. 2022a
*T.subregularis*	VPapp-GF2103	OL331097	OL314548	Unpublished
*T.subsinensis*	LWZ 20190611-9	OM523497	OM339304	Liu et al. 2022a
*T.subsphaerospora*	KHL 8511	AF347080	–	Unpublished
*T.termitophila*	AMO396	MN701025	MN687983	de Meiras-Ottoni et al. 2021
*T.termitophila*	AMO893	MN701026	MN687984	de Meiras-Ottoni et al. 2021
*T.torrendii*	URM85886	MK515148	MH280004	Unpublished
*T.tropica*	LWZ 20170613-16	OM523503	OM339311	Liu et al. 2022a
*T.tuberculata*	Dai17433	OM523507	OM339314	Liu et al. 2024a
*T.wenshanensis*	CLZhao 11649	OQ241389	OQ282716	This study
*T.wenshanensis*	CLZhao 11715	PP712100	–	This study
*T.wenshanensis*	CLZhao 22940	PP712101	–	This study
*T.yunnanensis*	CLZhao 210	NR177488	MN654918	Xu et al. 2019
*T.yunnanensis*	CLZhao 214	MN654922	MN654919	Xu et al. 2019

The sequences were aligned in MAFFT version 7 (Katoh et al. 2019) using the G-INS-i strategy. The alignment was adjusted manually using AliView version 1.27 (Larsson 2014). Each dataset was aligned separately at first and then the ITS+nLSU regions were combined with Mesquite version 3.51. The combined dataset was deposited in TreeBASE (submission ID 31349). Sequences of *Fibrodontiaalba* Yurchenko & Sheng H. Wu and *F.brevidens* (Pat.) Hjortstam & Ryvarden retrieved from GenBank were used as an outgroup in the ITS analysis (Luo and Zhao 2022).

Maximum parsimony analysis in PAUP* version 4.0a169 (http://phylosolutions.com/paup-test/) was applied to ITS+nLSU following a previous study (Zhao and Wu 2017). All characters were equally weighted and gaps were treated as missing data. Trees were inferred using the heuristic search option with TBR branch swapping and 1,000 random sequence additions. Max-trees were set to 5,000, branches of zero length were collapsed and all parsimonious trees were saved. Clade robustness was assessed using bootstrap (BT) analysis with 1,000 pseudo replicates (Felsenstein 1985). Descriptive tree statistics - tree length (TL), composite consistency index (CI), composite retention index (RI), composite rescaled consistency index (RC) and composite homoplasy index (HI) - were calculated for each maximum parsimonious tree generated. The combined dataset was also analysed using Maximum Likelihood (ML) in RAxML-HPC2 through the CIPRES Science Gateway (Miller et al. 2012). Branch support (BS) for the ML analysis was determined by 1000 bootstrap pseudoreplicates.

MrModeltest 2.3 (Nylander 2004) was used to determine the best-ﬁt evolution model for each dataset for the purposes of Bayesian inference (BI), Bayesian inference was performed using MrBayes 3.2.7a with a GTR+I+G model of DNA substitution and a gamma distribution rate variation across sites (Ronquist et al. 2012). A total of four Markov chains were run for two runs from random starting trees for 1.7 million generations for ITS+nLSU with tree and parameters sampled every 1,000 generations. The ﬁrst quarter of all of the generations were discarded as burn-ins. A majority rule consensus tree was computed from the remaining trees. Branches were considered as significantly supported if they received a maximum likelihood bootstrap support value (BS) of > 70%, a maximum parsimony bootstrap support value (BT) of > 70% or a Bayesian posterior probability (BPP) of > 0.95.

## ﻿Results

### ﻿Molecular phylogeny

**Figure 1.**
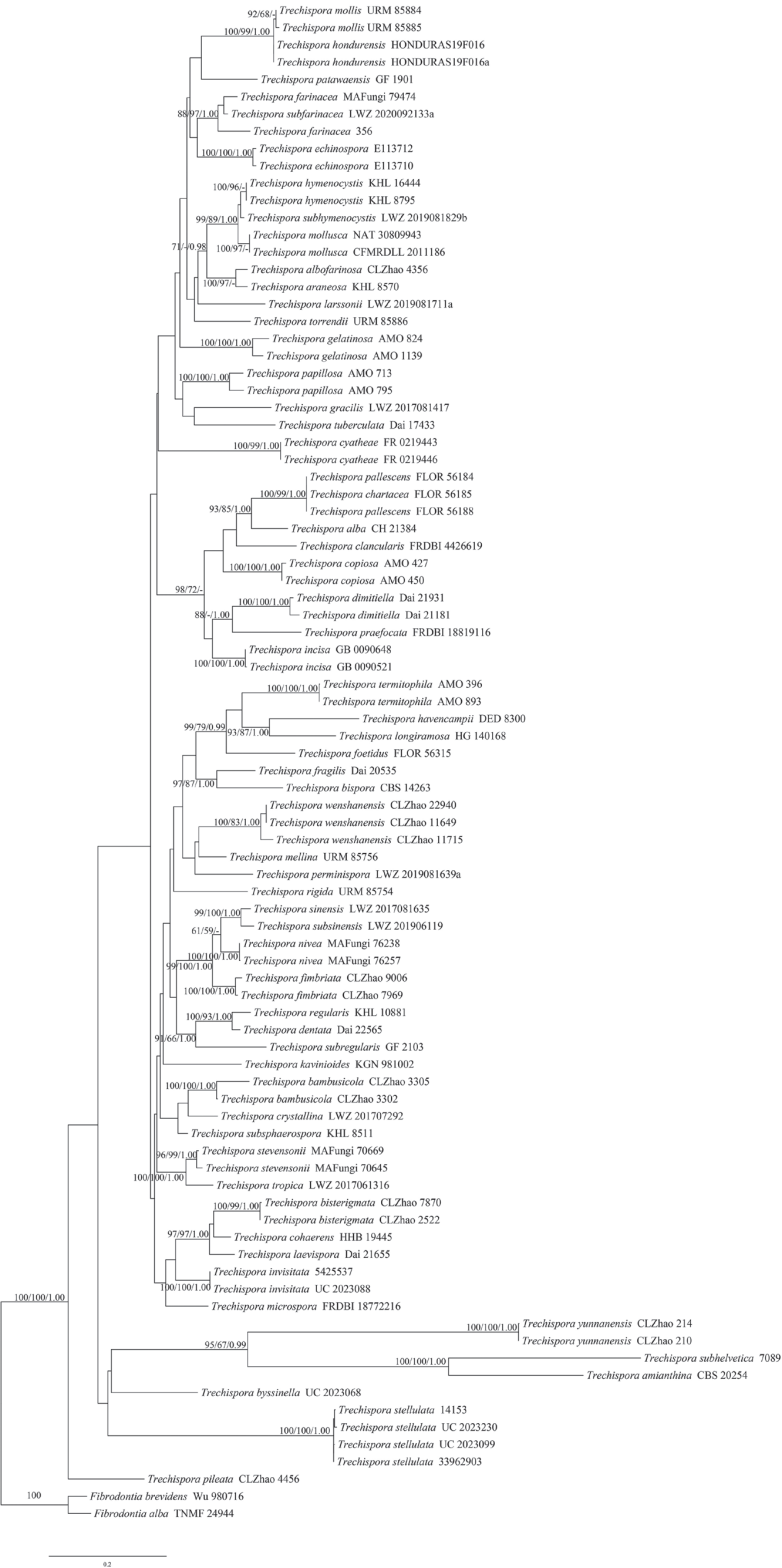
Maximum parsimony strict consensus tree illustrating the phylogeny of the four new species and related species in *Trechispora*, based on ITS+nLSU sequences. Branches are labelled with maximum likelihood bootstrap values > 70%, parsimony bootstrap values > 50% and Bayesian posterior probabilities > 0.95, respectively.

The ITS+nLSU dataset comprised sequences from 88 fungal specimens representing 64 taxa. The dataset had an aligned length of 2271 characters, of which 1376 characters were constant, 190 were variable and parsimony-uninformative and 705 were parsimony-informative. Maximum parsimony analysis yielded 300 equally parsimonious tree (TL = 5543, CI = 0.2979, HI = 0.7021, RI = 0.5278 and RC = 0.1572). The best model of nucleotide evolution for the ITS+nLSU dataset estimated and applied in the Bayesian analysis was found to be GTR+I+G. Bayesian analysis and ML analysis resulted in a similar topology as in the MP analysis. The Bayesian analysis had an average standard deviation of split frequencies = 0.012925 (BI) and the effective sample size (ESS) across the two runs is double the average ESS (avg. ESS) = 389. The phylogenetic tree inferred from the ITS+nLSU sequences highlighted that four new species were grouped into the genus *Trechispora* (Fig. 1).

### ﻿Taxonomy

#### ﻿ Trechispora albofarinosa

Taxon classificationFungiTrechisporalesHydnodontaceae

K.Y. Luo & C.L. Zhao sp. nov.

78592219-B023-5EAB-B455-E14D43E3390C

849463

Figs 2 , 3

##### Holotype.

China. Yunnan Province, Pu’er, Jingdong County, Huangcaoling, Wuliangshan National Nature Reserve, 24°23′N, 100°45′E, altitude 2350 m a.s.l., on the fallen branch of *Pinus*, leg. C.L. Zhao, 5 October 2017, CLZhao 4356 (SWFC).

**Figure 2.**
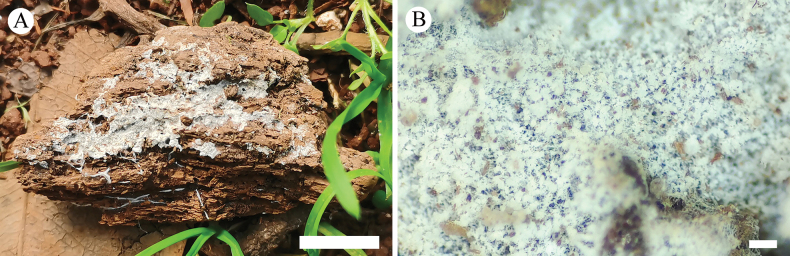
Basidiomata of *Trechisporaalbofarinosa* (holotype) **A** the front of the basidiomata **B** characteristic hymenophore. Scale bars: 1 cm **(A)**; 1 mm **(B)**.

**Figure 3.**
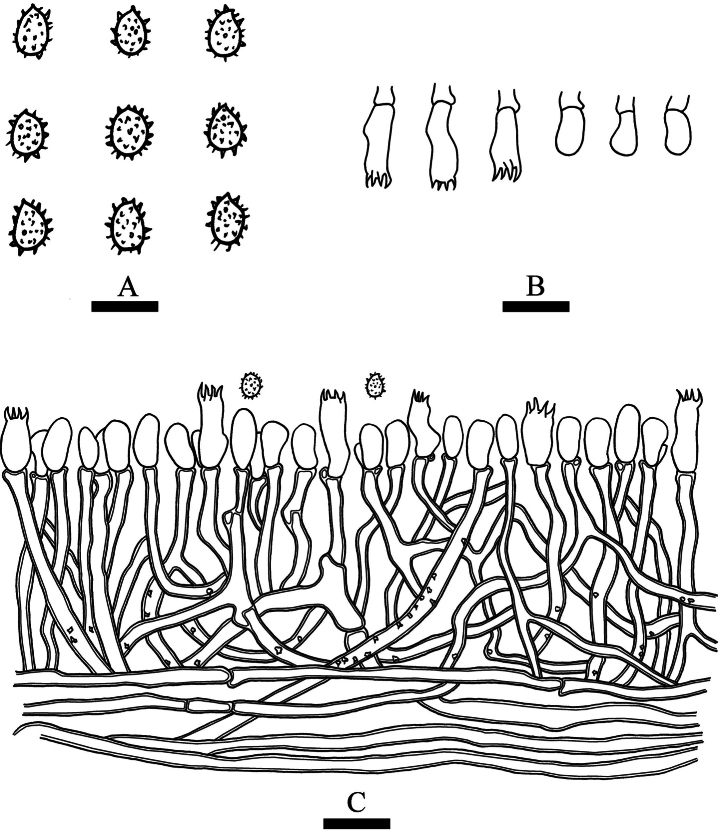
Microscopic structures of *Trechisporaalbofarinosa* (holotype) **A** basidiospores **B** basidia and basidioles **C** a cross section of basidiomata. Scale bars: 5 μm **(A)**; 10 µm **(B, C)**.

##### Etymology.

*Albofarinosa* (Lat.): referring to the farinose basidiomata with white hymenial surface.

##### Description.

Basidiomata annual, resupinate, farinose, without odor or taste when fresh, up to 3.5 cm long, 1.5 cm wide, and 300–500 µm thick. Hymenial surface flocculence, white when fresh, white to cream on drying. Sterile margin indistinct, white, and up to 0.5 mm wide.

Hyphal system monomitic, generative hyphae with clamp connections with ampullaceous septa, colorless, thick-walled, frequently branched, interwoven, 2–3.5 µm in diameter; IKI–, CB–, tissues unchanged in KOH.

Cystidia and cystidioles absent; basidia clavate, with four sterigmata and a basal clamp connection, 6.5–10 × 3.5–5 µm.

Basidiospores ellipsoid, colorless, thin-walled, aculeate, IKI–, CB–, 2.5–3.5 (–4) × 2–2.5 (–3.5) μm, L = 3.18 µm, W = 2.44 µm, Q = 1.3 (n = 30/1).

#### ﻿ Trechispora bisterigmata

Taxon classificationFungiTrechisporalesHydnodontaceae

K.Y. Luo & C.L. Zhao sp. nov.

67084F42-FD0F-5E84-95FD-13814F380BC2

849464

Figs 4 , 5

##### Holotype.

China. Yunnan Province, Yuxi, Xinping County, Mopanshan National Forestry Park, 23°56′N, 101°29′E, altitude 2200 m a.s.l., on the trunk of *Albiziajulibrissin*, leg. C.L. Zhao, 20 Aguest 2017, CLZhao 2522 (SWFC).

**Figure 4.**
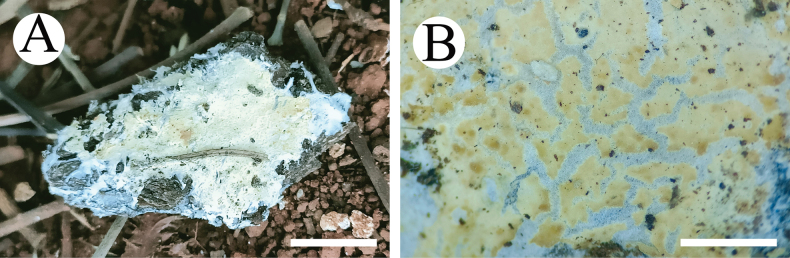
Basidiomata of *Trechisporabisterigmata* (holotype) **A** the front of the basidiomata **B** characteristic hymenophore. Scale bars: 1 cm **(A)**; 1 mm **(B)**.

**Figure 5.**
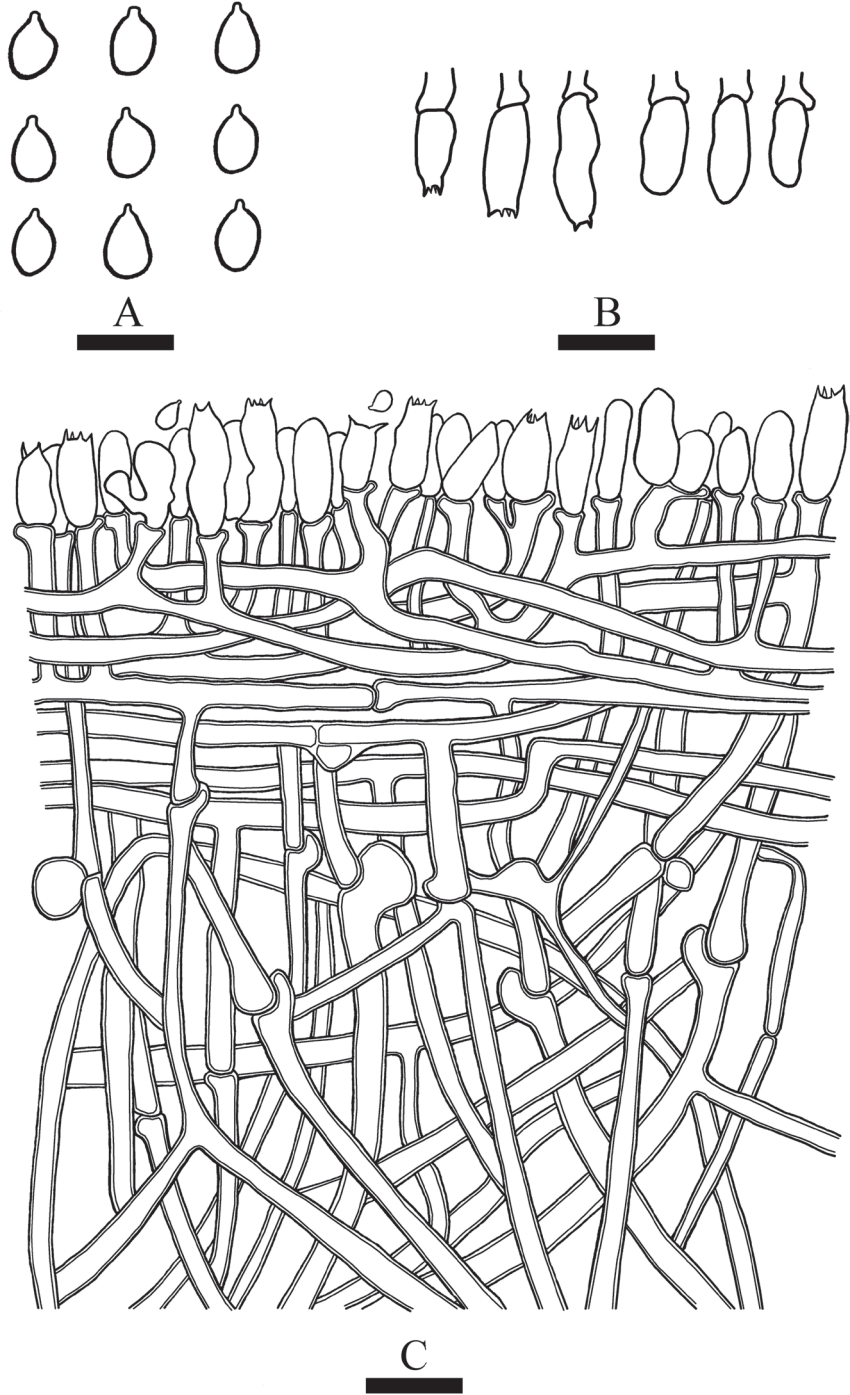
Microscopic structures of *Trechisporabisterigmata* (holotype) **A** basidiospores **B** basidia and basidioles **C** a cross section of basidiomata. Scale bars: 5 μm **(A)**; 10 µm **(B, C)**.

##### Etymology.

*Bisterigmata* (Lat.): referring to the basidia mainly with two sterigmata.

##### Description.

Basidiomata annual, resupinate, adnate, membranous, without odor or taste when fresh, up to 2.5 cm long, 1.5 cm wide, and 4 mm thick. Hymenial surface odontioid, cream. Sterile margin indistinct, white, rhizomorphic, up to 0.5 mm wide.

Hyphal system monomitic, generative hyphae with clamp connections, colorless, slightly thick-walled, ampullate septa frequently present in subiculum and hymenium with crystals, up to 6 µm wide, branched, interwoven, 2.5–4 µm in diameter; IKI–, CB–, tissues unchanged in KOH.

Cystidia and cystidioles are absent; basidia barrelled, slightly constricted, with two or four sterigmata and a basal clamp connection, 6.5–14.5 × 3.5–5.5 µm.

Basidiospores subglobose to broad ellipsoid, colorless, slightly thick-walled, smooth, IKI–, CB–, (2–) 2.5–4 × 2–3.5 µm, L = 3.03 µm, W = 2.41 µm, Q = 1.23–1.28 (n = 60/2).

##### Additional specimen examined

**(paratype).** China. Yunnan Province, Yuxi, Xinping County, Mopanshan National Forestry Park, 23°56′N, 101°29′E, altitude 2200 m a.s.l., on the living angiosperm tree, leg. C.L. Zhao, 19 August 2018, CLZhao 7870 (SWFC).

#### ﻿ Trechispora pileata

Taxon classificationFungiTrechisporalesHydnodontaceae

K.Y. Luo & C.L. Zhao sp. nov.

9DEDC498-5181-5D74-99D9-06F6C2A80180

849465

Figs 6 , 7

##### Holotype.

China. Yunnan Province, Pu’er, Jingdong County, Wuliangshan National Nature Reserve, 24°23′N, 100°45′E, altitude 2350 m a.s.l., on the angiosperm trunk, leg. C.L. Zhao, 6 October 2017, CLZhao 4456 (SWFC).

**Figure 6.**
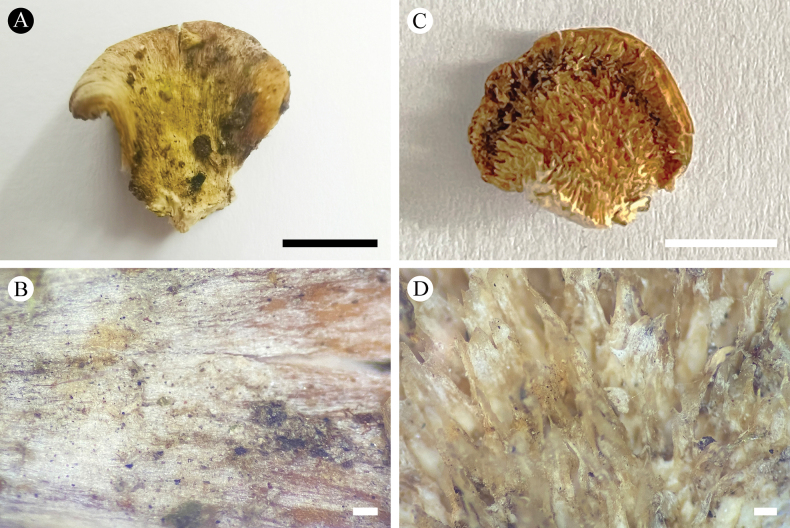
Basidiomata of *Trechisporapileata* (holotype) **A, B** the front of the basidiomata **C, D** the back of the basidiomata. Scale bars: 0.5 cm **(A)**; 1 mm **(B)**; 0.5 cm **(C)**; 1 mm **(D)**.

**Figure 7.**
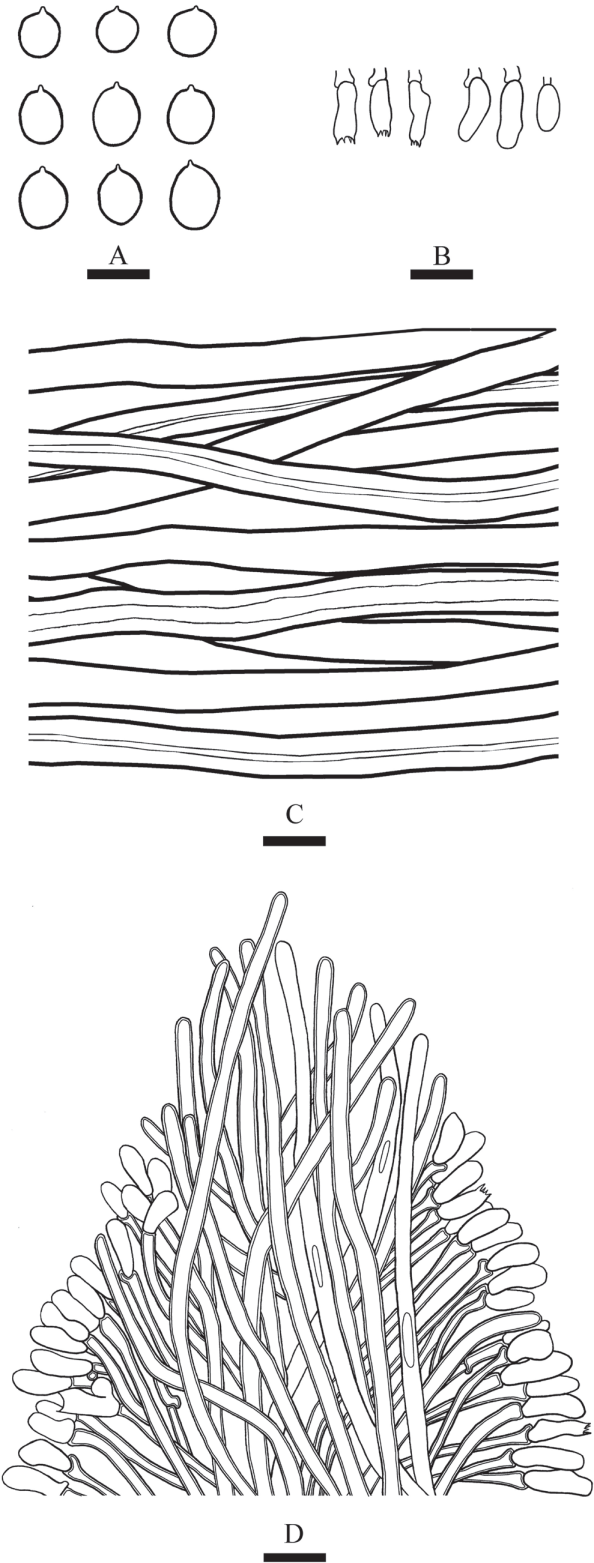
Microscopic structures of *Trechisporapileata* (holotype) **A** basidiospores **B** basidia and basidioles **C** hyphae of context of pileus **D** a spine trama of basidiomata. Scale bars: 5 μm **(A)**; 10 µm **(B, C)**.

##### Etymology.

*Pileata* (Lat.): referring to the pileate basidiomata.

##### Description.

Basidiomata annual, with a laterally contracted base, solitary or imbricate. Pileus fan shaped, cortical to corky, up to 1.5 cm long, 1 cm wide, and 2 mm thick, yellowish to yellowish brown, the surface radially striate covered with appressed scales, azonate; the hymenophore surface odontioid, yellowish brown, up to 1 mm long. Context cream, 1 mm thick. Sterile margin indistinct, slightly buff, and 0.5 mm wide.

Hyphal system monomitic, generative hyphae with clamp connections, colorless, thick-walled, frequently branched, interwoven, hyphae in spines 2.5–4 µm in diameter, IKI–, CB–, tissues unchanged in KOH. Hyphae in context colorless, thin- to thick-walled, unbranched, interwoven, 4.5–6 µm in diameter, IKI–, CB–, tissues unchanged in KOH.

Cystidia and cystidioles absent; basidia subcylindrical, constricted, with four sterigmata and a basal clamp connection, 5–7 × 2.5–4 µm.

Basidiospores subglobose to broad ellipsoid, colorless, thin-walled, smooth, IKI–, CB–, (2.5–) 2.8–5 (–5.5) × (2.5–) 3–4.7 µm, L = 4 µm, W = 3.56 µm, Q = 1.12 (n = 30/1).

#### ﻿ Trechispora wenshanensis

Taxon classificationFungiTrechisporalesHydnodontaceae

K.Y. Luo & C.L. Zhao sp. nov.

BC73DE38-7B6E-58FE-9B51-39937A54420B

Figs 8 , 9

##### Holotype.

China. Yunnan Province, Wenshan, Babao Town, Balao battle site, 23°22′N, 104°15′E, altitude 1300 m a.s.l., on the fallen angiosperm branch, leg. C.L. Zhao, 19 January 2019, CLZhao 11649 (SWFC).

**Figure 8.**
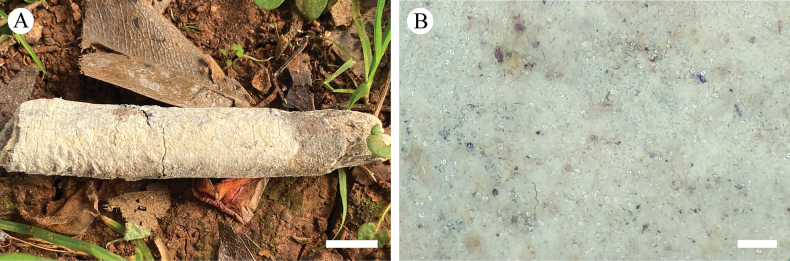
Basidiomata of *Trechisporawenshanensis* (holotype) **A** the front of the basidiomata **B** characteristic hymenophore. Scale bars: 1 cm **(A)**; 1 mm **(B)**.

**Figure 9.**
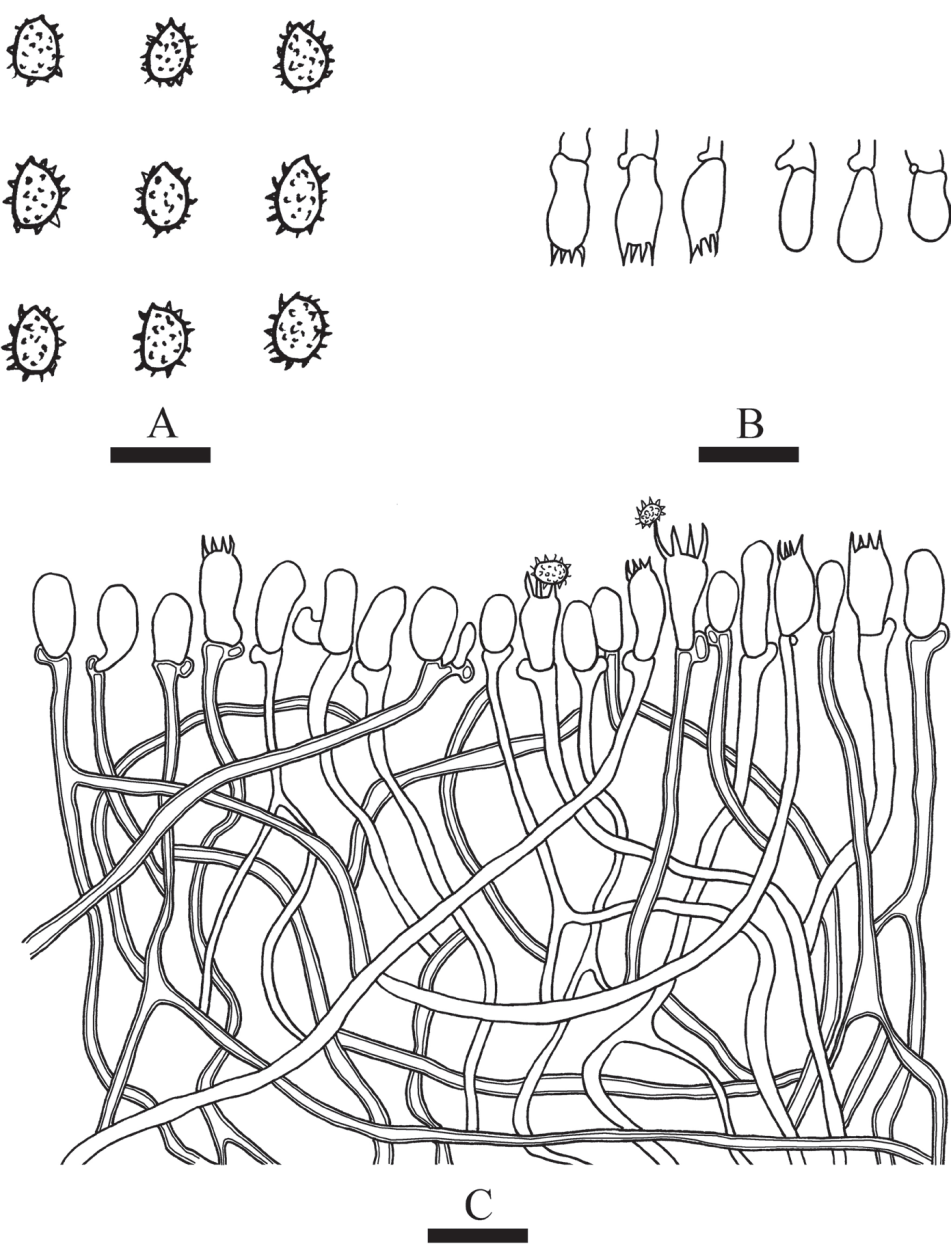
Microscopic structures of *Trechisporawenshanensis* (holotype) **A** basidiospores **B** basidia and basidioles **C** a cross section of basidiomata. Scale bars: 5 μm **(A)**; 10 µm **(B, C)**.

##### Etymology.

*Wenshanensis* (Lat.): referring to the locality (Wenshan) of the type specimen.

##### Description.

Basidiomata annual, resupinate, adnate, cottony, easily to separate from substrate, without odor or taste when fresh, up to 5.5 cm long, 4 cm wide, and 200–400 µm thick. Hymenial surface smooth, slightly cream when fresh, cream to buff on drying. Sterile margin indistinct, cream, and 1–2 mm wide.

Hyphal system monomitic, generative hyphae with clamp connections, colorless, thin- to thick-walled, branched, interwoven, 1–2 µm in diameter; IKI–, CB–, tissues unchanged in KOH.

Cystidia and cystidioles are absent; basidia barrelled, with four sterigmata and a basal clamp connection, 7–10 × 3–5 μm.

Basidiospores ellipsoid, colorless, thin-walled, warted, IKI–, CB–, (2–) 2.5–3.7 (–4) × (1.5–) 2–3 µm, L = 3.02 µm, W = 2.37 µm, Q = 1.25–1.30 (n = 90/3).

##### Additional specimens examined

**(paratypes).** China. Yunnan Province, Wenshan, Funing county, Guying village, 23°42′N, 105°53′E, altitude 1000 m a.s.l., on the fallen angiosperm branch, leg. C.L. Zhao, 20 January 2019, CLZhao 11715; Yunnan Province, Lincang, Lancangjiang Forestry Region, 25°37′N, 97°30′E, altitude 1750 m a.s.l., on the fallen angiosperm branch, leg. C.L. Zhao, 21 July 2022, CLZhao 22940 (SWFC).

## ﻿Discussion

Many recently described wood-inhabiting fungal taxa have been reported in the subtropics and tropics, including in the genus *Trechispora* (Ordynets et al. 2015; Phookamsak et al. 2019; Xu et al. 2019; Chikowski et al. 2020; Haelewaters et al. 2020; Crous et al. 2021; de Meiras-Ottoni et al. 2021; Zhao and Zhao 2021; Liu et al. 2022a, b; Luo and Zhao 2022; Deng et al. 2023; Sommai et al. 2023). The present study reports four new species in *Trechispora*, based on a combination of morphological features and molecular evidence.

Based on ITS+nLSU topology (Fig. 1), four new species were grouped into the genus *Trechispora*, in which *T.albofarinosa* was sister to *T.araneosa* (Höhn. & Litsch.) K.H. Larss., However, morphologically, *T.araneosa* can be delimited from *T.albofarinosa* by its odontioid to poroid hymenial surface and larger basidiospores (5–6.5 × 4–5 µm; Larsson 1995). The second new species *T.bisterigmata* grouped closely with *T.cohaerens* (Schwein.) Jülich & Stalpers and *T.laevispora* Z.B. Liu, Y.D. Wu & Yuan Yuan. However, morphologically, *T.cohaerens* is different from *T.bisterigmata* by its thin-walled hyphal (Jülich and Stalpers 1980); *T.laevispora* can be delimited from *T.bisterigmata* by having the smooth hymenial surface, and thin-walled basidiospores (Liu et al. 2022b). The third species *T.pileata* formed a monophyletic lineage. The species *T.wenshanensis* grouped closely with *T.mellina* (Bres.) K.H. Larss. However, morphologically, *T.mellina* can be delimited from *T.wenshanensis* by having the longer basidia (15–20 × 4.5–5 µm) and smooth basidiospores (Chikowski et al. 2020).

Morphologically, *Trechisporaalbofarinosa* resembles *T.olivacea* K.Y. Luo & C.L. Zhao and *T.yunnanensis* C.L. Zhao by sharing the farinosa basidiomata. However, *T.olivacea* differs from *T.albofarinosa* by olivaceous hymenial surface and thick-walled basidiospores (Luo and Zhao 2022); *T.yunnanensis* can be delimited from *T.albofarinosa* due to its thick-walled, larger basidiospores (7–8.5 × 5–5.5 µm; Xu et al. 2019). The new species *T.albofarinosa* is similar to *T.bambusicola* C.L. Zhao, *T.fimbriata* C.L. Zhao, *T.fissurata* C.L. Zhao and *T.murina* K.Y. Luo & C.L. Zhao in its presence of ellipsoid basidiospores. *T.bambusicola* can be delimited from *T.albofarinosa* by odontioid hymenial surface with aculei cylindrical to conical (0.3–0.5 mm long), and thick-walled basidiospores (Zhao and Zhao 2021); *T.fimbriata* can be delimited from *T.albofarinosa* due to its hydnoid hymenial surface, and thick-walled basidiospores (Zhao and Zhao 2021); *T.fissurata* is different from *T.albofarinosa* by hydnoid hymenial surface and thick-walled, broadly basidiospores (3.3–4 × 2.8–3.5 µm; Zhao and Zhao 2021); *T.murina* can be delimited from *T.albofarinosa* due to its grandinioid hymenial surface and thick-walled basidiospores (Luo and Zhao 2022).

*Trechisporabisterigmata* is similar to *T.fastidiosa* (Pers.) Liberta by sharing the membranous basidiomata. However, *T.fastidiosa* differs from *T.bisterigmata* by smooth hymenial surface and larger basidiospores (6–7 × 4.5–5.5 µm; Bernicchia and Gorjón 2010). *T.bisterigmata* resembles *T.bambusicola* C.L. Zhao, *T.canariensis* Ryvarden & Liberta and *T.christiansenii* (Parmasto) Liberta in its monomitic hyphal system and presence of the crystals. However, *T.bambusicola* differs from *T.bisterigmata* by its odontioid hymenial surface and ornamented basidiospores (Zhao and Zhao 2021); *T.canariensis* differs from *T.bisterigmata* due to its larger basidia (15–20 × 5–6 μm) and thin-walled, larger basidiospores (5–7 × 3–3.5 μm; Ryvarden and Liberta 1978); *T.christiansenii* can be delimited from *T.bisterigmata* by its larger basidia (15–20 × 6–7 μm) and larger basidiospores (5.5–7 × 4–4.5 μm; Liberta 1966).

*Trechisporapileata* is similar to *T.byssinella* (Bourdot) Liberta, *T.kavinioides* B. de Vries, *T.silvae*-*ryae* (J. Erikss. & Ryvarden) K.H. Larss. and *T.subsphaerospora* (Litsch.) Liberta by sharing smooth basidiospores. However, *T.byssinella* differs from *T.pileata* by having narrower ellipsoid basidiospores (Bernicchia and Gorjón 2010); *T.kavinioides* can be delimited from *T.pileata* by its odontioid hymenial surface, and narrower ellipsoid to lacrymiform basidiospores (Bernicchia and Gorjón 2010); *T.silvae*-*ryae* is different from *T.pileata* by dimitic hyphal system (Bernicchia and Gorjón 2010); *T.subsphaerospora* differs from *T.pileata* by having angular basidiospores (Bernicchia and Gorjón 2010). In addition, the delimitation characteristics of the genus have full resupinate basidiomata, but this new species has the pileate basidiomata with a laterally contracted base. Based on the phylogenetic analyses, this new species groups with *Trechispora* species, therefore, we propose that the genus *Trechispora* accommodate this new species in the present study.

*Trechisporawenshanensis* resembles *T.fastidiosa* and *T.laevispora* Z.B. Liu, Y.D. Wu & Yuan Yuan by sharing a smooth hymenial surface. However, *T.fastidiosa* differs from *T.wenshanensis* by larger basidiospores (6–7 × 4.5–5.5 µm; Bernicchia and Gorjón 2010); *T.laevispora* differs from *T.wenshanensis* by fimbriate margin of the basidiomata and smooth basidiospores (Liu et al. 2022b). The new species *T.wenshanensis* is similar to *T.bambusicola* C.L. Zhao, *T.fimbriata* C.L. Zhao, *T.fissurata* C.L. Zhao, *T.murina* K.Y. Luo & C.L. Zhao and *T.yunnanensis* C.L. Zhao due to its ellipsoid basidiospores. However, *T.bambusicola* can be delimited from *T.wenshanensis* by odontioid hymenial surface, and thick-walled basidiospores (Zhao and Zhao 2021); *T.fimbriata* differs from *T.wenshanensis* due to its hydnoid hymenial surface, and thick-walled basidiospores (Zhao and Zhao 2021); *T.fissurata* is different from *T.wenshanensis* by hydnoid hymenial surface, and thick-walled, broadly basidiospores (3.3–4 × 2.8–3.5 µm; Zhao and Zhao 2021); *T.murina* can be delimited from *T.wenshanensis* due to its grandinioid hymenial surface, and thick-walled basidiospores (Luo and Zhao 2022); *T.yunnanensis* is different from *T.wenshanensis* by farinaceous hymenial surface and thick-walled, larger basidiospores (7–8.5 × 5–5.5 µm; Xu et al. 2019).

### ﻿Key to 42 accepted species of *Trechispora* in China

**Table:** 

1	Basidiomata with clavarioid	**2**
–	Basidiomata without clavarioid	**6**
2	Basidiomata grayish brown to pale purple	**3**
–	Basidiomata pure white to pale yellow	**4**
3	Basidiomata with dense branches and long terminal branches	***T.longiramosa***
–	Basidiomata with loose branches	***T.laxa***
4	Basidiomata with flattened branches	**5**
–	Basidiomata without flattened branches	***T.tongdaoensis***
5	Basidiomata branches polychotomous	***T.alba***
–	Basidiomata branches dichotomous	***T.khokpasiensis***
6	Basidiomata pileate	***T.pileata***
–	Basidiomata resupinate to effused	**7**
7	Hymenophore poroid	**8**
–	Hymenophore smooth, colliculose, irpicoid, grandinioid, odontioid, hydnoid	**13**
8	Hyphal system dimitic	***T.dimitiella***
–	Hyphal system monomitic	**9**
9	Subicular hyphae thick-walled	**10**
–	Subicular hyphae thin-walled	**11**
10	Ampullate septa present on subicular hyphae	***T.mollusca***
–	Ampullate septa absent on subicular hyphae	***T.suberosa***
11	Crystals in subiculum as numerous rodlets	***T.candidissima***
–	Crystals in subiculum as rhomboidal plates or various shapes	**12**
12	Sphaerocysts present in cords and the adjacent part of subiculum	***T.hymenocystis***
–	Sphaerocysts absent	***T.subhymenocystis***
13	Basidiospores smooth	**14**
–	Basidiospores ornamented	**16**
14	Basidiomata with rhizomorph	***T.bisterigmata***
–	Basidiomata without rhizomorph	**15**
15	Basidiospores subglobose, angular to turbinate	***T.confinis***
–	Basidiospores ellipsoid	***T.laevispora***
16	Basidiomata < 50 µm thick	**17**
–	Basidiomata > 50 µm thick	**19**
17	Crystals absent	***T.gracilis***
–	Crystals present	**18**
18	Crystals aggregated, rhomboidal fakes	***T.perminispora***
–	Crystals butterfly-like, easily broken into irregular shapes	***T.subaraneosa***
19	Hymenophore smooth	**20**
–	Hymenophore colliculose, irpicoid, grandinioid, odontioid, hydnoid	**27**
20	Basidiospores slightly cyanophilous	***T.incisa***
–	Basidiospores acyanophilous	**21**
21	Basidiospores > 6.5 µm long	***T.yunnanensis***
–	Basidiospores < 6.5 µm long	**22**
22	Generative hyphae < 2 µm in diameter	***T.wenshanensis***
–	Generative hyphae > 2 µm in diameter	**23**
23	Generative hyphae thin-walled	**24**
–	Generative hyphae thick-walled	**25**
24	Hymenophore farinaceous	***T.larssonii***
–	Hymenophore arachnoid	***T.subfarinacea***
25	Generative hyphae > 3.5 µm in diameter	***T.latehypha***
–	Generative hyphae < 3.5 µm in diameter	**26**
26	Basidiospores ellipsoid, thin-walled	***T.albofarinosa***
–	Basidiospores broadly ellipsoid to globose, thick-walled	***T.olivacea***
27	Hymenial surface colliculose, irpicoid or grandinioid	**28**
–	Hymenial surface odontioid or hydnoid	**30**
28	Generative hyphae thick-walled	***T.murina***
–	Generative hyphae thin-walled	**29**
29	Growth on bamboo	***T.taiwanensis***
–	Growth on other plant	***T.crystallina***
30	Tramal hyphae thin-walled or slightly thick-walled	**31**
–	Tramal hyphae distinctly thick-walled	**35**
31	Crystals absent in trama	***T.tropica***
–	Crystals present in trama	**32**
32	Basidiospores subglobose to globose	***T.odontioidea***
–	Basidiospores ellipsoid or broadly ellipsoid	**33**
33	Tramal hyphae 3–6 µm wide, spines of basidiospores constricted	***T.constricta***
–	Tramal hyphae 2–4 µm wide, spines of basidiospores not constricted	**34**
34	Cystidia present	***T.chaibuxiensis***
–	Cystidia absent	***T.nivea***
35	Hymenophore aculei > 0.4 mm long	**36**
–	Hymenophore aculei < 0.4 mm long	**39**
36	Margin smooth	***T.fissurata***
–	Margin fimbriate	**37**
37	Basidiomata irpicoid	***T.dentata***
–	Basidiomata odontioid or hydnoid	**38**
38	Hymenophore aculei sparse, cream to buff-yellow when fresh	***T.fimbriata***
–	Hymenophore aculei dense, white when fresh	***T.fragilis***
39	Generative hyphae ampullate septa absent	***T.bambusicola***
–	Generative hyphae ampullate septa present	**40**
40	Basidiospores with sharp spines	***T.subfissurata***
–	Basidiospores without sharp spines	**41**
41	Spines of basidiospores constricted	***T.subsinensis***
–	Spines of basidiospores not constricted	***T.sinensis***

## Supplementary Material

XML Treatment for Trechispora albofarinosa

XML Treatment for Trechispora bisterigmata

XML Treatment for Trechispora pileata

XML Treatment for Trechispora wenshanensis

## ﻿Additional information

### Author ORCIDs

Jiang-Qing Su https://orcid.org/0009-0008-5480-4502

Chang-Lin Zhao https://orcid.org/0000-0002-8668-1075

### Data availability

All of the data that support the findings of this study are available in the main text.
